# Outcomes of Unilateral Inferior Oblique Myectomy Surgery in Inferior Oblique Overaction Due to Superior Oblique Palsy

**DOI:** 10.4274/tjo.02170

**Published:** 2016-01-05

**Authors:** Erhan Yumuşak, Ümit Yolcu, Murat Küçükevcilioğlu, Oktay Diner, Fatih Mehmet Mutlu

**Affiliations:** 1 Kırıkkale University Faculty of Medicine, Department of Ophthalmology, Kırıkkale, Turkey; 2 Sarıkamış Military Hospital, Ophthalmology Clinic, Kars, Turkey; 3 Gülhane Military Medical Academy, Department of Ophthalmology, Ankara, Turkey

**Keywords:** Inferior oblique myectomy, inferior oblique overaction, superior oblique palsy

## Abstract

**Objectives:**

To present the outcomes of unilateral inferior oblique myectomy performed in patients with inferior oblique overaction due to superior oblique palsy.

**Materials and Methods:**

Twenty-seven eyes of 27 patients that underwent inferior oblique myectomy surgery for superior oblique palsy between 2002 and 2008 were included. Inferior oblique overaction scores (between 0-4) at preoperative, early postoperative (within 1 week after surgery) and late postoperative (earliest 6 months) visits were reviewed.

**Results:**

There were 12 male and 15 female patients. Eighteen were operated on the right eye, and 9 were operated on the left eye. The mean age was 15.62±13.31 years, and the mean follow-up was 17±11.28 months (range, 6-60 months). Patients who had horizontal component and V-pattern deviation were excluded. Preoperative and early postoperative inferior oblique overaction scores were 2.55±0.75 and 0.14±0.36, respectively, and the difference was statistically significant (p<0.01). This improvement was maintained up to the late postoperative period.

**Conclusion:**

Due to its promising short-term and long-term results, inferior oblique myectomy can be the first choice of surgery for inferior oblique overaction due to superior oblique palsy.

## INTRODUCTION

Superior oblique palsy (SOP) is the most frequent single extraocular muscle paralysis diagnosed by ophthalmologists.^1^ Due to the torsional component and the frequency of incomitance, medical treatment using prisms is generally not tolerated by SOP patients. The condition is commonly treated with surgery.

The first surgical approaches for the inferior oblique (IO) muscle date back to the mid 19th century. In 1841, John Taylor made an incision into the outer edge of the IO and probably performed tenotomy on the IO in order to treat strabismus patients. Duanne was the first to begin safely and deliberately performing IO surgery in 1905.^2^ Until 1943, myectomy was the only IO surgery performed. White^3^ and Brown^4^ later described the recession procedure. The transposition approach for IO surgery was first reported in 1982 by Elliott and Nankin.^5^ Prior to that, Parks^6^ showed that recession was more effective than myectomy.

Inferior oblique muscle overaction (IOOA) was classified by Parks^6^ into two groups: primary and secondary. Secondary IOOA is associated with ipsilateral SOP, whereas in primary IOOA there is no SOP or contralateral superior rectus palsy. Primary IOOA is frequently bilateral and usually asymmetrical, making it difficult to choose a surgical option. In these cases, overaction is generally greater in the non-fixating, amblyopic eye, though a very small, even imperceptible amount may be found in the fixating eye. V-pattern is typical. This type of overaction does not spontaneously resolve. Chamberlain^7^ recommended asymmetric bilateral surgery for these cases. He showed that after a unilateral weakening surgery for the eye with more overaction, secondary IOOA is unmasked in the fellow eye in 37% of cases.

In this study, we present the outcomes of unilateral IO myectomy in patients with SOP-associated IOOA.

## MATERIALS AND METHODS

Twenty-seven eyes of 27 patients who underwent unilateral IO myectomy due to unilateral SOP at the Gülhane Military Medical Academy Department of Ophthalmology between 2002 and 2008 were evaluated retrospectively. Patients with bilateral or masked SOP were excluded from the study. Preoperative, early postoperative (within 1 week of surgery), and late postoperative (at least 6 months after surgery) IOOA scores (on a scale of 0-4) were analyzed.^8^ Patients who were followed for at least 6 months after surgery were included in the study.

Forced traction test was conducted preoperatively under topical anesthesia for patients aged 20 years or older. For patients under 20 years old, the test was done under general anesthesia immediately before the surgery.

In the forced traction test, toothed forceps were used to grasp the eye near the limbus at the 6 and 12 o’clock positions and check whether the eye moved left and right freely. If eye movement was limited, the test was considered positive and the patient was excluded from the study.

All surgeries were performed under general anesthesia after the forced traction test. An incision was made in the inferotemporal conjunctiva 6 mm from and parallel to the limbus. Blunt and sharp dissection were used to expose the IO muscle. The IO was suspended with a fine muscle hook and separated from the insertion site. A length of approximately 5 mm was resected, and the muscle ends were released after establishing hemostasis. The conjunctiva was closed with 8/0 silk suture.

Statistical Package for the Social Sciences version 16.0 software was used for statistical analyses. A t-test was used to compare pre- and postoperative IOOA and the initial and final postoperative follow-up results. Level of statistical significance was accepted as α=0.05.

## RESULTS

The study included 12 male patients and 15 female patients. The mean age of the patients was 15.62±13.31 years (range, 2-59 years) and the mean follow-up time was 17±11.28 months (range, 6-60 months). Unilateral-ipsilateral myectomy was performed in all cases. The right eye was operated in 18 patients and the left eye in 9 patients. Patients with horizontal component and V-pattern deviation were excluded from the study. Descriptive statistics of the patients includedin the study are shown in Table 1.

The patients’ mean preoperative IOOA grade was 2.55±0.75. In the early postoperative period the mean IOOA grade was 0.14±0.36, and this difference was statistically significant (p<0.01). Postoperative IOOA severity remained stable throughout follow-up for most of the patients; however, 2 patients’ IOOA grade increased from 0 to 1 in the final follow-up and 2 other patients’ IOOA grade decreased from 1 to 0. Cumulatively, there was no statistically significant difference in IOOA severity between the early and late postoperative periods (p>0.05) (Table 2).

Fifteen of the 27 patients (55.5%) had abnormal head position (AHP) preoperatively. After surgery, AHP was corrected in 12 of those 15 patients (80%).

## DISCUSSION

IOOA is an incomitant vertical deviation characterized by eye elevation caused by adduction. Its etiology is categorized as primary or secondary. Although mechanical and innervational causes are suspected to act in the etiology of primary IOOA, the cause is not fully understood. It frequently accompanies horizontal deviation, but it can also occur in isolation. It usually does not lead to vertical deviation in primary position. It may be unilateral or bilateral, symmetrical or asymmetrical. Secondary IOOA develops due to weakness of the ipsilateral superior oblique muscle or the contralateral superior rectus muscle. These patients may exhibit vertical or cyclovertical deviation in primary position and AHP.

SOP is the most common isolated extraocular muscle paralysis and is usually treated with surgery. It is most commonly congenital or idiopathic (63%), though head trauma, cerebrovascular disorders, tumors, sinusitis and myasthenia gravis have also been reported as etiologic factors. The most important symptoms of SOP are hypertropia, extorsion, AHP and diplopia.^9,10^

Both primary and secondary IOOA have been successfully treated with myectomy, recession and transposition procedures for many years. Ghazawy et al.^11^ evaluated the early and late postoperative outcomes of myectomy and transposition surgeries in 120 eyes of 81 patients irrespective of etiology. The two procedures had very similar outcomes, but they recommended myectomy, which is a shorter and easier procedure. Their myectomy results (mean postoperative IOOA was 0.29) were quite similar to those in our clinic.

Soyugelen et al.^12^ evaluated the outcomes of IO myectomies in a total of 28 patients, 18 unilateral and 10 asymmetric. The mean follow-up duration was 15 months; the mean pre-and postoperative IOOA severity was 2.88±0.75 and 0.16±0.38, respectively. The asymmetric cases had pre- and postoperative IOOA severity of 4.40±0.69 and 2.00±0.94, respectively. Patients with a horizontal component were not included in our study, but the results we obtained with isolated myectomy are comparable.

Recession is another surgical procedure used to treat IOOA. In a study by Rajavi et al.^8^ including 82 eyes of 50 patients, eyes randomly underwent myectomy (42 eyes) or recession (40 eyes). They were unable to detect a statistically significant difference in the outcomes of the two procedures. Min et al.^13^ conducted a prospective study in which 20 children with bilateral +3 IOOA underwent myectomy in one eye and anterior transposition in the fellow eye and were followed for 20 months. They detected recurrence starting in the first month in myectomized eyes, whereas they detected no major changes during the 20 month follow-up period in the eyes that underwent anterior transposition. Because the success criterion accepted for the study was zero IOOA, success rates of 25% for the myectomy group and 85% for the anterior transposition group were achieved after 20 months. In other words, Min et al.^13^ stated that at the end of 20 months, the success of the myectomies had progressively decreased, whereas there was no marked change in the anterior transposition group. We did not observe such outcomes with myectomy. However, Min et al.^13^ accepted +1 IOOA as failure criteria, although this grade of IOOA has been evaluated in many studies in the literature as subclinical and asymptomatic. Therefore, like many other clinical studies, our results are not consistent with those of Min et al.^13^ in terms of change over time or success criteria.^12,14,15,16,17^

AHP is one of the important diagnostic criteria of SOP. Bahl et al.^18^ compared IO myectomy and recession surgeries in SOP cases and found no differences between the two surgical procedures in terms of AHP. In their study, 49% of the myectomy patients had AHP preoperatively, and approximately 53% of those patients’ ABP improved postoperatively. Elliott and Nankin^5^ reported an ABP correction rate of 88% after IO anterotransposition surgery. Of the patients in our study, 55.5% had AHP prior to surgery, and AHP improved after surgery in 80% of those cases. We believe that the varying rates of AHP correction reported in the literature can be attributed to differences in diagnosis, age groups, surgical technique and success criteria.

The most important complications in IO myectomy are adherence syndrome, which results from the free ends adhering to the sclera or one another, and IOOA recurrence.^4^ Furthermore, if hemostasis is not achieved, severe bleeding can occur both during and after surgery, which can affect success in the early and late postoperative periods. We did not encounter these types of complications in any of our cases.

## CONCLUSION

In conclusion, in the treatment of IOOA, myectomy is a procedure that can be completed quickly. As scleral sutures are not required, it carries no risk of scleral perforation, and several possible complications can be easily avoided with good hemostasis. Due to its satisfactory results in both the short and long terms, myectomy can be the first choice of surgical method for cases of IOOA due to SOP.

## Ethics

Ethics Committee Approval: It was taken, Informed Consent: It was taken, Peer-review: Externally peer-reviewed.

## Figures and Tables

**Table 1:**
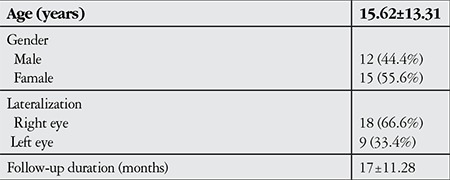
Demographic and clinical characteristics of the patients (n=27)

**Table 2:**
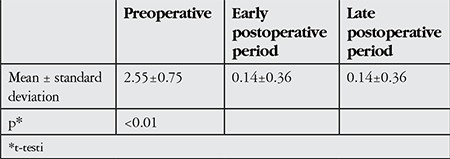
Comparison of pre- and postoperative inferior oblique overaction severity
